# Strategic spatiotemporal vaccine distribution increases the survival rate in an infectious disease like Covid-19

**DOI:** 10.1038/s41598-020-78447-3

**Published:** 2020-12-09

**Authors:** Jens Grauer, Hartmut Löwen, Benno Liebchen

**Affiliations:** 1 Institut für Theoretische Physik II: Weiche Materie, Heinrich-Heine-Universität Düsseldorf, 40225 Düsseldorf, Germany; 2 Institut für Festkörperphysik, Technische Universität Darmstadt, 64289 Darmstadt, Germany

**Keywords:** Statistical physics, thermodynamics and nonlinear dynamics, Physics

## Abstract

Present hopes to conquer the Covid-19 epidemic are largely based on the expectation of a rapid availability of vaccines. However, once vaccine production starts, it will probably take time before there is enough vaccine for everyone, evoking the question how to distribute it best. While present vaccination guidelines largely focus on individual-based factors, i.e. on the question to whom vaccines should be provided first, e.g. to risk groups or to individuals with a strong social-mixing tendency, here we ask if a strategic spatiotemporal distribution of vaccines, e.g. to prioritize certain cities, can help to increase the overall survival rate of a population subject to an epidemic disease. To this end, we propose a strategy for the distribution of vaccines in time and space, which sequentially prioritizes regions with the most new cases of infection during a certain time frame and compare it with the standard practice of distributing vaccines demographically. Using a simple statistical model we find that, for a locally well-mixed population, the proposed strategy strongly reduces the number of deaths (by about a factor of two for basic reproduction numbers of $R_0\sim 1.5-4$ and by about 35% for $R_0\sim 1$). The proposed vaccine distribution strategy establishes the idea that prioritizing individuals not only regarding individual factors, such as their risk of spreading the disease, but also according to the region in which they live can help saving lives. The suggested vaccine distribution strategy can be tested in more detailed models in the future and might inspire discussions regarding the importance of spatiotemporal distribution rules for vaccination guidelines.

## Introduction

The Covid-19 pandemic 2019/2020^1–5^ has led to more than 40 million infections and 1 million deaths worldwide (October 2020)^6,7^ and an unprecedented social and economic cost which comprises a sudden rise of the number of unemployments by more than 20 million in the USA alone, and a damage of trillions of dollars at the stock market and in the worldwide real economy^8^. This situation challenges politicians to decide on suitable measures and researchers to explore their efficiency, based on models allowing to forecast and compare the evolution of infectious diseases (like Covid-19) when taking one or the other action.

Available measures to efficiently deal with epidemic outbreaks at low infection numbers include a rigorous contact-tracing (e.g. based on “Corona-Apps”^9^) and -testing combined with quarantine of infected individuals^10–13^. Strict travel restrictions preventing an infectious disease from entering disease-free regions (or to die out locally^14^) present an alternative measure^15,16^, whereas travel reductions by less than $\sim 99\%$^17^ slow down the spreading of the disease only slightly^17–19^.

At higher infection numbers, the only way to avoid an explosion of contagions is to reduce the contact rate through measures that largely influence the everyday life of the population, such as social distancing^11,13,20–23^ and lock-down^13,24^. If a population does not persistently reduce the contact rate to the point where infection rates decrease (this requires a contact reduction of $>60\%$ for a basic reproduction number of $R_0=2.5$^22^), the majority of its members must endure the disease—until it finally reaches herd immunity^25^.

The main hope which remains at such stages rests on the rapid discovery and admission of vaccine^26,27^ (or antibodies^28^) to accelerate reaching herd immunity. However, while every day where an infectious disease like Covid-19 is active may cause thousands of additional deaths, even after admission, it may take months until sufficient vaccine is available to overcome an infectious disease. Therefore it is important to strategically distribute the available vaccines such that the number of deaths remains as small as possible. Surprisingly, both official vaccination guidelines, e.g. for pandemic influenza^29,30^, and previous works on vaccine distribution^31–33^, focus on the question to whom vaccine should be mainly provided, e.g. to prioritize individuals by age or disease risk, and leave the quest for a suitable spatial and temporal vaccine distribution aside. (Other works like^34^ ask for the optimal vaccine production rate.) This results in the common practice of simply distributing vaccines proportionally to the population density^35^.

**Figure 1:**
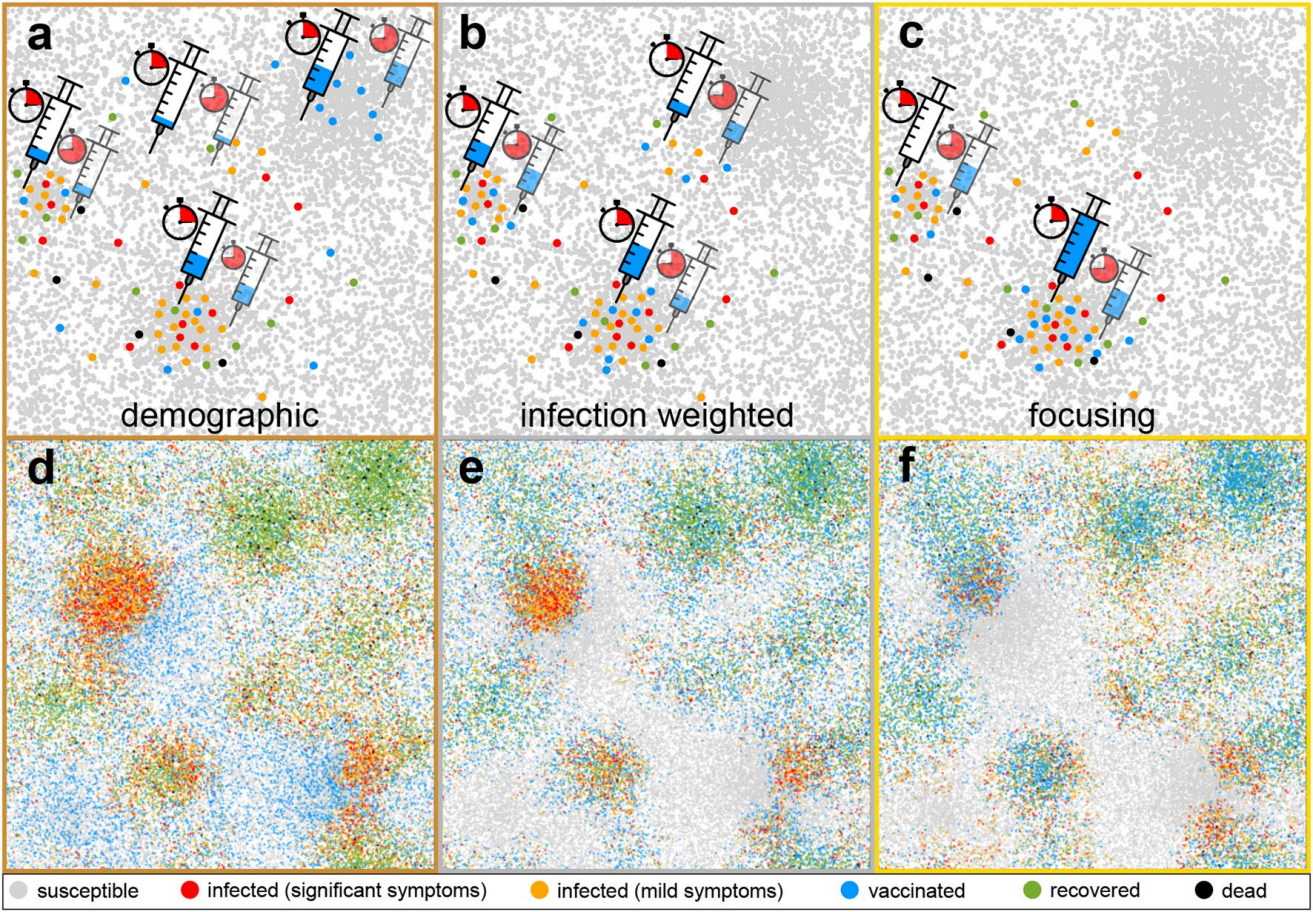
Schematic illustration of the proposed spatiotemporal vaccine distribution strategies and of the simulation model. (**a**) shows the standard “demographic strategy”, where vaccines (dosage needles) are continuously distributed among all regions (e.g. cities) proportionally to their population density (dots represent groups of individuals). (**b**) shows the “infection weighted” strategy, where vaccines are distributed proportionally to the local bi-linear incidence rates (red and orange dots) and (**c**) shows the “focusing strategy” where at early times (clocks; transparent syringes show the vaccine distribution at later times) only the region with the largest bi-linear incidence rate receives vaccines, until the rate of a second region catches up and also receives vaccines. (**d**)–(**f**) show typical simulation snapshots for an inhomogeneously distributed population with a “city size distribution” following Zipf’s law, taken 56 days after the onset of vaccination when following the demographic strategy, the infection weighted strategy or the focusing strategy, respectively. The legend below shows the states in our model.

In the present work we propose alternative strategies for the spatiotemporal distribution of gradually produced vaccines, which hinge on the idea that the number of deaths due to a spreading infectious disease is controlled by the bi-linear incidence rate $\beta SI$^36^, which increases linearly in the number of susceptibles *S* and infections *I*, with $\beta$ being the transmission coefficient, not by population density. With the “infection weighted strategy” (see Fig. 1b,e), the available vaccine is distributed proportionally to the calculated bi-linear incidence rate. This strategy can be further improved by sequentially prioritizing the regions (cities) with the highest bi-linear incidence rate, and correspondingly the highest number of new infections in a certain time frame (see Fig. 1c,f and the Supplementary Movie); that is by exclusively providing, or “focusing”, all available vaccines to those regions (“focusing strategy”). To compare the infection weighted and focusing strategy with the “demographic” vaccine distribution practice, we develop a simple statistical model describing the time-evolution of an epidemic outbreak (such as Covid-19) and its response to vaccination. As our central result, we find that the number of deaths resulting from infections occurring after the onset of vaccine production is *generally* lower, i.e. for the all considered initial reproduction numbers ($R_0\sim 1-4$) and vaccine production rates as well as in the absence and in the presence of additional social distancing rules, when following the focusing strategy rather than the demographic distribution practice. In fact, for sufficiently inhomogeneous infection patterns, the focusing strategy reduces the number of deaths by more than a factor of two, for a large range of basic reproduction numbers $R_0$ and vaccine production rates. The difference is largest for $R_0\sim 2-3$, i.e. it features a peak in this range, as might be typical for Covid-19 if no additional measures are in action, but even for $R_0\sim 1$ the focusing strategy significantly increases the survival probability.

**Figure 2:**
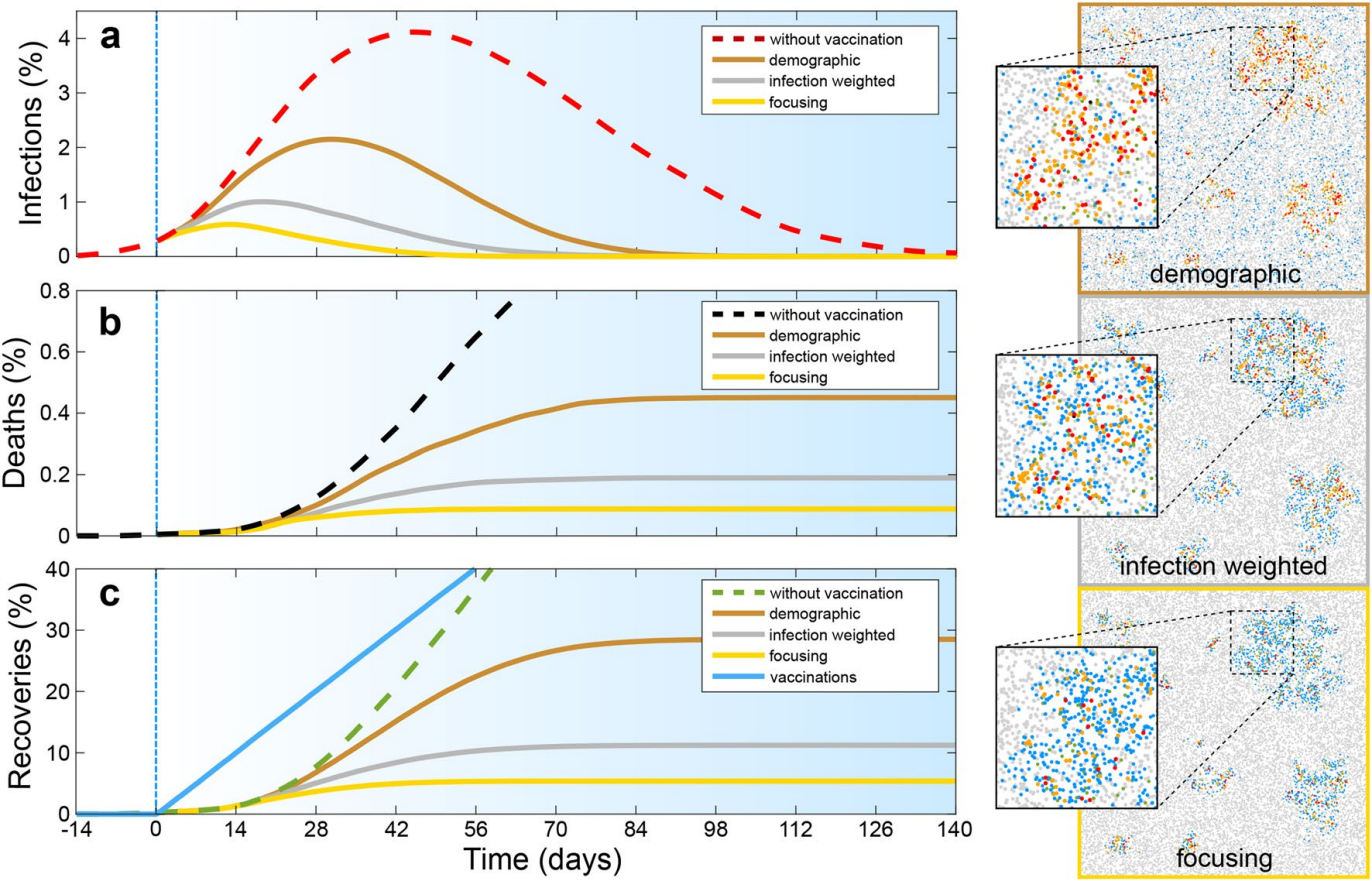
Competition of spatiotemporal vaccine distribution strategies regarding the time evolution of the fraction of infected individuals (**a**), the fraction of deaths (**b**), and of recoveries and vaccinations (**c**). Dashed red lines show simulation results without vaccination and bronze, silver (or grey) and gold show results for the demographic vaccine distribution strategy, the infection weighted strategy and the focusing strategy respectively. The blue line in panel (**c**) shows the vaccinated fraction of the population and vertical blue lines mark the onset of vaccination; the specific time of which is unimportant (see text). Panels on the right show simulation snapshots taken 14 days after the onset of vaccine production; insets magnify extracts of these snapshots. Parameters: Disease duration $t_D=14 \; \text {days}$; latency time $t_L=t_D/3$, survival probability $s_r=0.965, s_o=0.99$, total vaccination rate $\nu =0.1N/t_D$ and initial reproduction number $R_0=2.5$. (The latter is based on $D=10^2 R_c^2/t_D$, $\beta _o=0.3$, $\beta _r=0.1$; see “Methods”); $L=500R_c$; curves are averaged over 100 random initial ensembles with $N=6000$.

## Model

To explore the impact of the spatiotemporal vaccine distribution on the disease-evolution in detail, we now introduce a computational model, which is based on Brownian agents and allows deriving a (nonuniform) statistical mean-field model as we will discuss below. Both models are expected to apply to situations where the population is *locally* well-mixed. The model describes the dynamics of *N* agents moving randomly in continuous space in a box of size $L\times L$ with periodic boundary conditions. The agents represent groups of individuals and have an internal state variable, which is inspired by the SIR model^37–39^ and its variants^40–44^. We use colors (see legend in Fig. 1) to represent the possible states in our simulations, which refer to individuals which are “susceptible” (grey), “infected with weak symptoms” (orange), “infected with significant symptoms” (red), “recovered” (green) and “vaccinated” (blue). Infected agents (orange and red) have an inner clock; they remain symptom free for a latency time $t_L$ and then show mild (orange) or significant (red) symptoms for a duration $t_D-t_L$. After an overall disease duration of $t_D$ they either recover with a survival probability $s_{o,r}$ (green) or die with probability $1-s_{o,r}$ (black), where the indices refer to agents with mild (orange) and significant symptoms (red), respectively. To model the infection dynamics we describe the spatial motion of an agent with position $\mathbf{r}_i(t)$ using Brownian dynamics $\dot{\mathbf{r}}_i(t)=\sqrt{2D}\varvec{\eta }_i(t)$, where *D* is the diffusion coefficient controlling how fast agents move and $\varvec{\eta }_i(t)$ represents Gaussian white noise with zero mean and unit variance. We assume that all infected agents (orange and red) are infectious, both in the latent phase and afterwards (as for Covid-19) and infect a fraction of $\beta _o + \beta _r$ of those susceptible agents (grey) which are closer than a distance $R_c$; here, indices refer to mild (orange) and significant (red) symptoms. Agents showing significant symptoms (red) do not move but can infect “visitors” if actively approaching them.

To connect the suggested model with standard mean-field descriptions for infectious diseases, we now deduce a continuum model from the Langevin equations describing the agent dynamics. The resulting model can be viewed as a generalization of standard mean-field models such as the SIR and the SEIR model to inhomogeneous situations and cases where mild and strong infections coexist (as for Covid-19). Let us now consider continuous variables (fields) representing the local mean number density of susceptible agents $S(\mathbf{r},t)$, exposed agents $E(\mathbf{r},t)$ (infected but not yet diseased), infected agents which are free of symptoms (or have mild symptoms) $F(\mathbf{r},t)$, infected agents with symptoms $I(\mathbf{r},t)$, recovered (immune) agents $R (\mathbf{r},t)$ and victims $V(\mathbf{r},t)$. In the absence of social forces (pair attractions, social distancing), the following equations follow by translating Langevin equations to Smoluchowski equations^45^ and coupling them via suitable reaction terms:

$$\begin{aligned} \dot{S}(\mathbf{r},t)&= - \beta ' (E+F+I) S/\rho _0 + D \nabla ^2 S - \nabla \cdot (S \mathbf{f}) - \nu ' \\ \dot{E}(\mathbf{r},t)&= \beta ' (E+F+I) S/\rho _0 - \alpha E + D \nabla ^2 E - \nabla \cdot (E \mathbf{f}) \\ \dot{F} (\mathbf{r},t)&= \alpha r E - \delta F + D\nabla ^2 F - \nabla \cdot (F \mathbf{f}) \\ \dot{I}(\mathbf{r},t)&= \alpha (1-r) E - \delta I - \nabla \cdot (I \mathbf{f}) \\ \dot{R} (\mathbf{r},t)&= \delta (s_o F+ s_r I) + D \nabla ^2 R - \nabla \cdot (R \mathbf{f}) + \nu ' \\ \dot{V}(\mathbf{r},t)&= \delta (1-s_r) I + \delta (1-s_o) F \end{aligned}$$

Note here that the exposed state explicitly shows up as a dynamical variable at the continuum level, but only implicitly in our agent-based simulations where infected agents have an inner clock and are in the latent phase before showing (mild) symptoms. In the above equations, $\beta '$ is the effective contact rate, i.e. $1/\beta '$ is the mean time between infectious contacts; $\alpha =1/t_L$ is the rate to switch from the exposed (latent) state to the infected state, $\delta = 1/(t_D-t_L)$ is the recovery rate and $\nu '(\mathbf{r},t)$ is the spatiotemporal vaccination rate which is linked to the constant total vaccination rate in the agent-based model via $\nu =\int \mathrm{d}{} \mathbf{r} \; \nu '(\mathbf{r},t)$. The number *r* is the ratio of infections proceeding symptom free (or with mild symptoms) and $\rho _0=N/L^2$ is the mean agent density. Finally, *D* is the diffusion coefficient and $\mathbf{f}(\mathbf{r}) = -\nabla _{\mathbf{r}} U/\gamma$ is the reduced force due to the external potential which we use to create a density profile mimicking a typical city size distribution. The overall density converges to a Boltzmann distribution $S+E+F+I+R+V = N \mathrm{exp}[-U(\mathbf{r})/(kT)]/ \int \mathrm{exp}[-U(\mathbf{r})/(kT)] \mathrm{d} \mathbf{r}$, yielding the conservation law $\int (S+E+F+I+R+V)\; \mathrm{d} \mathbf{r} =N$ which can be viewed as an expression of the conservation of the overall number density (or the number of agents) in the coarse of the dynamics.

Numerically solving this model by using finite difference simulations now allows us to further test the spatiotemporal vaccination strategies. In our simulations we start with the initial state $E=F=R=V=0$ and $S=1-\epsilon$, $I=\epsilon$ where $\epsilon (\mathbf{r},t)$ represents a small perturbation of the unstable steady state (e.g. $E=F=I=R=V=0, S=1$ for $U=0$), which represents the population before the emergence of the disease. The results of these simulations confirm that the spatiotemporal distribution of continously distributed vaccines plays an important role; also here, the infection-weighted strategy and the focusing strategy strongly increase the number of survivors as compared to the demographic distribution.

## Results

We now perform numerical simulations of both the proposed agent based model and the statistical mean-field model which both lead to consistent results. For the agent based model we perform Brownian dynamics simulations^46–51^ starting with $2 \times 10^{-3} N$ randomly distributed initial infections and an initial reproduction number $R_0=2.5$ such that infection numbers exponentially increase over time. Let us assume that vaccine production starts after some initial transient and then allows to transfer $\nu$ individuals per day from the susceptible to the immune state. (Note that the duration of the initial transient is unimportant in our simulations, if vaccination starts long before herd immunity is reached.) Now considering the time-evolution of the percentage of infected, dead and recovered individuals of a given population, and distributing the available vaccines proportionally to the population density (bronze curves in Fig. 2), we observe an infection maximum (panel a) about 30 days (two infection cycles) after the onset of vaccine production, i.e. when about 22% of the population have received vaccines and 2% of the population is infected. When distributing the available vaccines proportionally to the local bi-linear incidence rate $\beta SI$ instead, which according to $\dot{I} \propto \beta SI$ refers to the number of new infected cases in a given time frame (“infection weighted strategy”), notably, the infection maximum occurs an entire infection cycle earlier (silver curve in panel a). Here the infection number peaks when only 11% of the population has received vaccines and only 1% is infected. However, the infection weighted strategy is not optimal but can be further improved by exclusively providing all available vaccines to the region (e.g. a city) with the highest incidence rate (“focusing strategy”). This means that initially only a single region receives vaccines until the number of new infected individuals in a second region catches up and both regions simultaneously receive vaccines, until a third region catches up and so on. Following this “focusing strategy” the infection peak further shifts to earlier times (golden curve in panel a) and occurs when only 0.6% of the population is infected. Importantly, the resulting fraction of deaths reduces by more than a factor of two when following the infection weighted strategy (silver) rather than the demographic strategy (bronze). It almost halves again when following the focusing strategy instead (gold). This shows that the precise spatial and temporal order of vaccine donation controls the number of survivors from an infectious disease.

We now complement these results by numerical solutions of the statistical mean-field model equations by finite-difference simulations. As in our particle based simulations we find that the focusing strategy is generally better than the infection-weighted strategy and the demographic vaccine distribution strategy. The results of the agent-based simulations and the continuum simulations show a close quantitative agreement (not shown for the uniform system; see Fig. 4 for an exemplaric quantitative comparison in the presence of “cities”.).

**Figure 3:**
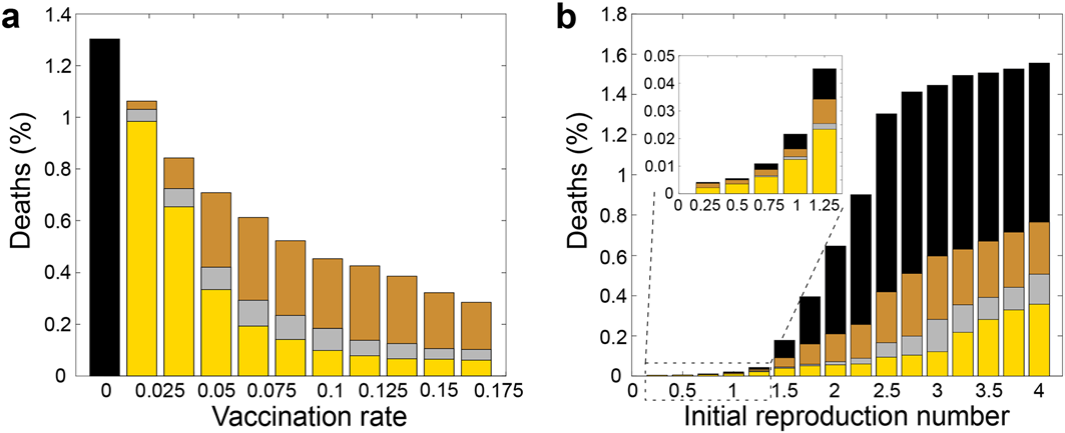
Fraction of deaths as a function of the vaccine production rate (left) and the initial basic reproduction number (right) for the demographic strategy (bronze), the infection-weighted strategy (silver) and the focusing strategy (gold). Results without vaccination (black) are shown for comparison. The results are based on the agent-based model; the statistical mean-field equations lead to very similar graphs. Parameters are shown in the key; remaining ones are as in Fig. 2.

**Figure 4:**
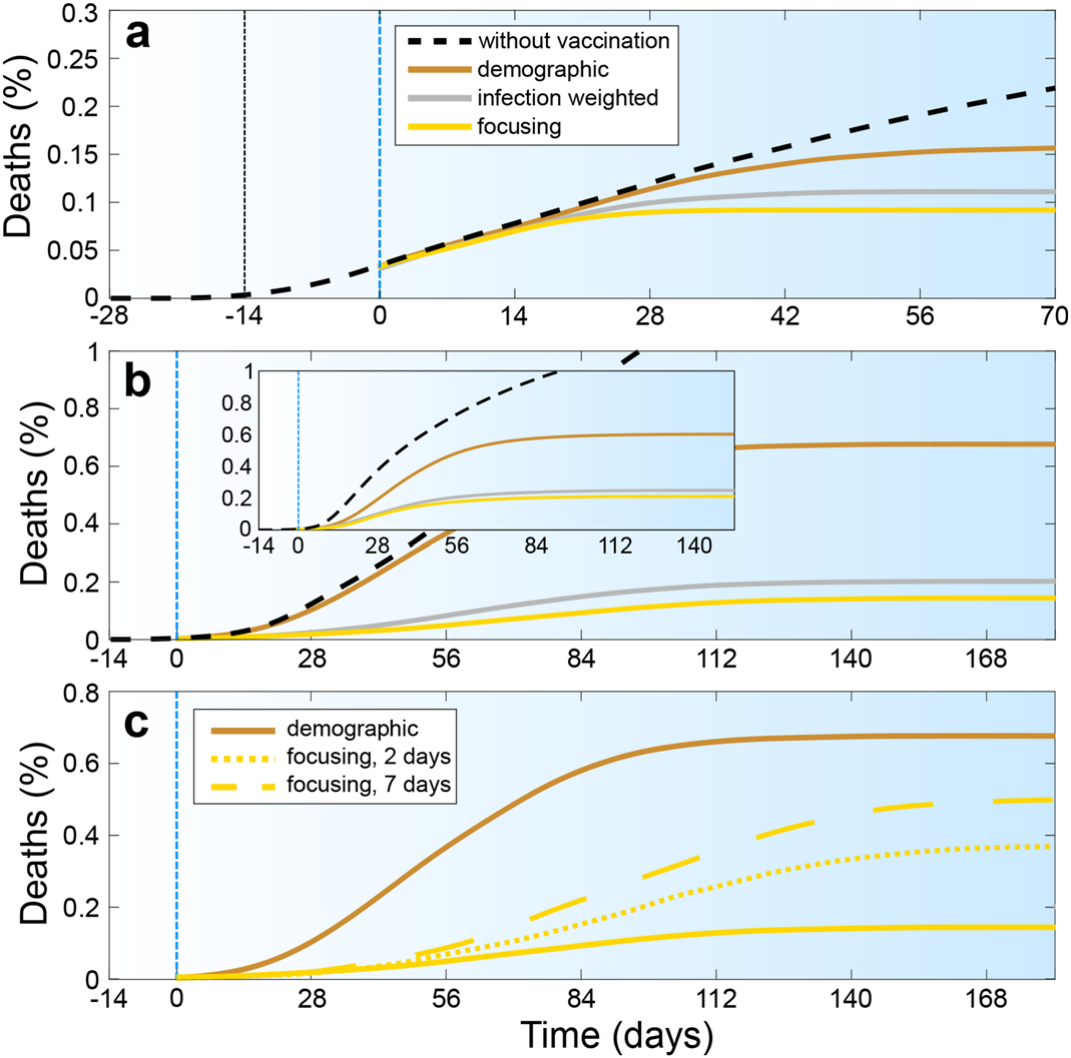
Competition of spatiotemporal vaccination strategies (**a**) in the presence of social distancing which is activated after 14 days (black vertical line) and reduces the reproduction number to $R\approx 1$ (**b**) for a population density distribution following Zipf’s law. Colors and parameters are as in Fig. 2 but we have $N=12000$, $L=700$, $R_0=2.7$ (which is based on $D=10^3 R_c^2/t_D$ and $\beta _o=0.05$, $\beta _r=0.017$) and $\nu =0.05N/t_D$. Inset: Analogous results for the mean-field model using same parameters as in the agent-based model and a 140 $\times$ 140-grid with each grid point corresponding to a spatial area of $5R_c \times 5R_c$ (**c**) assuming a delay of 2 (dotted golden curve) and 7 (dashed golden curve) days in the registration of the cases of infection. Parameters are as in Fig. 4b.

**Figure 5:**
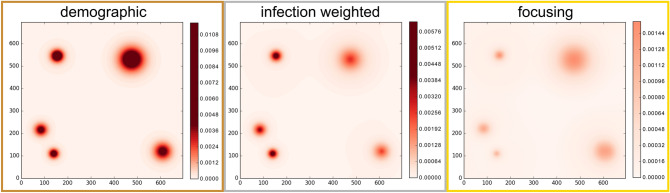
Snapshots of the infection patterns 56 days after the onset of vaccination, based on the statistical mean-field model. Colors show the density of exposed agents $E(\mathbf {r},t)$. Parameters are as in Fig. 4b.

To systematically explore the robustness of these findings we now repeat our simulations for different vaccine production rates and initial reproduction numbers. Figure  3 shows that the resulting fraction of deaths, counted once the disease is gone, is generally highest for the demographic strategy (bronze) and lowest for the focusing strategy (gold). Mathematically, this is because vaccination is most efficient at locations where it maximally inhibits the development of new cases of infections, which holds true independently of the specific parameter regime. The differences among the individual strategies is comparatively large if vaccine is produced fast enough to allow vaccinating at least about 1% of the population per day and at reproduction rates around $R_0\sim 2-3$. The latter value might be sensible for Covid-19. However, even for slower vaccine production or for $R_0\sim 1-2$ (as typical for influenza), several percent of deaths can be avoided in our simulations by strategically distributing the available vaccines in space and time.

To further explore the applicability-regime of the focusing strategy, we now combine it with social distancing rules, which reduce the effective reproduction number to $R_t\sim 1$. We implement the latter as a phenomenological repulsive three-body interaction among the agents (see “Methods” for details) which prevents them from aggregating in groups of more than two individuals. Also here, the resulting deaths fraction (Fig. 4a) saturates significantly earlier when following the focusing strategy (gold) rather than the demographic strategy (bronze). The difference in deaths numbers among the three different vaccination strategies is almost identical to our corresponding results at $R_0\sim 1$ but without social distancing (Fig.3b).

Finally, we explore a possible impact of a nonuniform population distribution (city structure) on the proposed vaccination strategies. We create a population with a spatial density distribution following Zipf’s law which closely describes the city size distribution in most countries^52^ as $\tilde{P}_c(s>S) \propto 1/S$, where $\tilde{P}_c(s)$ is the probability that a city is larger than *S*. To generate a population featuring a corresponding population distribution, we add an external potential *U* to the equation of motion of the agents (see “Methods” for details). Following statistical mechanics, the resulting population density follows Boltzmann’s law $P(\mathbf{r}) \propto \mathrm{exp}[-U(\mathbf{r})/(kT)]$ where $P(\mathbf{r})$ is the probability that an agent is at position $\mathbf{r}$ and $kT=\gamma D$ is the effective thermal energy of the agents, controlling how often agents leave a “city” (minimum of *U*). Now matching Boltzmann’s distribution with Zipf’s law yields a construction rule for *U* (see “Methods”) to create a population pattern featuring a characteristic city-size distribution. Our resulting simulations, shown in Fig. 4b, and in the Supplementary Movie (for $N=55.000$ agents), demonstrate that the focusing strategy and the infection weighted-strategy again halve the number of deaths compared to the demographic strategy. Here, the former two strategies are comparatively close to each other regarding the number of resulting deaths, which indicates that in strongly inhomogeneous populations a suitable spatial vaccine distribution rule might be even more important than the precise temporal sequence of vaccine donation. While in the previous simulations we assumed an immediate registration of infected persons, we have tested the strategies when the time scale for registration of infections is delayed by up to seven days (dashed and dotted golden curves in Fig. 4c). Even in the presence of such a delay, we obtain a reduced number of deaths when following the focusing strategy.

To further test the robustness of these findings, we have performed continuum simulations of our statistical mean-field model, which leads to close quantitative agreement with the particle based simulations (Fig. 4b). Typical snapshots of the infection pattern 56 days after the onset of vaccination are shown in Fig. 5. These figures show a clear reduction of the infection number in all infection hotspots for the focusing strategy (panel c) as compared to the infection weighted strategy (b) and in particular compared to the demographic vaccine distribution practice (a).

## Discussion

Our findings establish the idea that the optimal vaccine distribution depends not only on individual-based factors (who first) but also on the spatiotemporal distribution (e.g. where to provide vaccines first). In particular, our results have shown that by sequentially prioritizing spatial regions (cities) with the highest local bi-linear incidence rates, the proposed “focusing strategy” significantly reduces the number of deaths compared to the standard practice of distributing vaccines demographically. Specifically for locally well-mixed populations, initial reproduction numbers $R_0\sim 1.5-4$ and a sufficiently inhomgeneous infection pattern, and if vaccine production starts long before the population reaches herd immunity, our simulations reveal that the focusing strategy can reduce the number of deaths by more than a factor of two (and for $R_0 \sim 1$ by up to about $35\%$). These findings should be further tested in detailed models in the future e.g. to explore the impact of the proposed strategy also in situations where the population is not locally well-mixed and to combine the suggested spatiotemporal distribution strategy with individual-based factors such as the the prioritization of risk groups, individuals with a strong social mixing tendency or with jobs of systemic relevance. Finally, it should be noted that its applicability hinges on a reasonably detailed knowledge e.g. of the actual local infection numbers and the relevant delay times in the communication of tests.

## Methods

### Simulation details

To calculate the spatial dynamics of the agents in our model, we solve Langevin equations $\dot{\mathbf{r}}_i(t)=\sqrt{2D}\varvec{\eta }_i(t)$ with $i=1,..,N$ using Brownian dynamics simulations involving a forward Euler time-stepping algorithm and a time-step of $dt=0.0028$ days which amounts to about 4 minutes. After each timestep we check for each infected agent (red or orange) which susceptible agents (grey) are closer than $R_c$. We then change the state of the latter agents to an infected state with a transmission rate of ${\tilde{\beta }}_o=3{\tilde{\beta }}_r=0.0075/dt$ (Figs. 2, 3, 4a), corresponding to infections with mild symptoms (orange) and significant symptoms (red), respectively. These rates yield $\beta _o=3\beta _r=0.3$ for the corresponding fractions of contacts which lead to infections. See Table 1 for a list of the simulation parameters which we use in the present work.

**Table 1:** Typical simulation parameters.

Disease duration $t_D$	$14\, \text {days}$
Latency time $t_L$	$t_D/3$
Vaccination rate $\nu$	$0.1N/t_D$
Initial reproduction number $R_0$	2.5–3
Survival probability $s_r$	$96.5\%$
Survival probability $s_o$	$99\%$
Effective contact rate $\beta '$	$R_0/t_D$
Diffusion coefficient *D*	$10^2$–$10^3 R_c^2/t_D$
Number of agents *N*	6.000–55.000
Simulation box length *L*	500–$700R_c$
Strength of “city” potential *a*	$D\gamma /2$
“City radius” $R_{min},R_{max}$	$20,80R_c$

### Additional simulations

**Figure 6:**
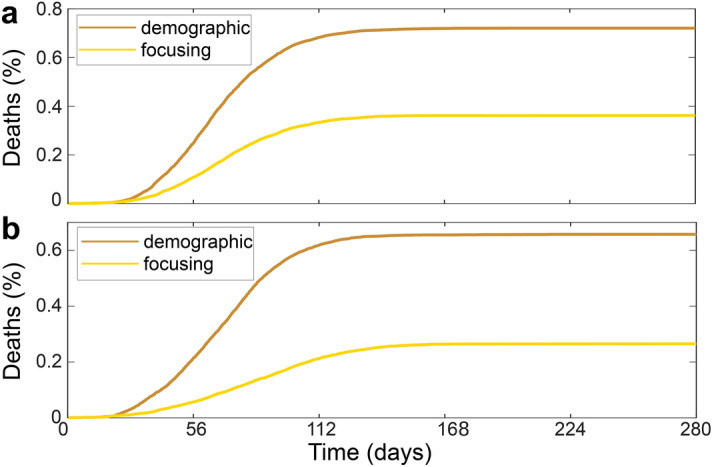
Fraction of deaths over time for (**a**) active particles with inertia and self-propulsion and (**b**) particles with different mobilities.

In order to demonstrate that the obtained results do not depend on the details of our simulations, but are rather to be understood as a generic outcome, we have performed additional simulations based on a different particle based model. In particular, we have investigated active underdamped particles, which feature inertia, unlike the Brownian agents considered in our model, and move in a box of size $L\times L$ with periodic boundary conditions. We have have also tested this with hard-wall boundary conditions and find similar results (not shown here). In these simulations each particle has an internal drive, represented by an effective self-propulsion force $\mathbf {F}_{SP,i}=\gamma _t v_0 \mathbf {u}(\theta _i)$, where $\mathbf {u}(\theta _i)=(cos(\theta _i),sin(\theta _i))$ is the direction of self-propulsion. The behavior of the particles with masses *m* and moments of inertia *I* is now substantially different and the underlying equations for the velocities $\mathbf {v}_i$ and orientations $\theta _i$ are

1 $$\begin{aligned} m \frac{d\mathbf{v}_i(t)}{dt}= & {} -\gamma _t \mathbf {v}_i - \nabla _{\mathbf{r}_i} U + \mathbf {F}_{SP,i} + \sqrt{2D}\gamma _t \varvec{\eta }_i , \end{aligned}$$

2 $$\begin{aligned} I \frac{d^2\theta _i(t)}{dt^2}= & {} -\gamma _r \frac{d\theta _i}{dt} + \sqrt{2D}\gamma _r \xi _i, \end{aligned}$$

where $\varvec{\eta}_i(t), \xi _i(t)$ represent Gaussian white noise of zero-mean unit variance and $\gamma _t,\gamma _r$ are are translational and rotational drag coefficients. In the simulations we again obtain a significantly reduced number of deaths when applying the focusing strategy as shown in Fig. 6a (with $m/\gamma _t=I/\gamma _r=10^3/t_D, v_0=50R_c/t_D$).

In addition, we carried out further simulations in which we tested the strategies in a structured population in which individuals differ from each other. For this purpose we assigned different mobilities to the agents and modeled two groups of individuals within the population, one with very low mobility ($D=5 \times 10^2 R_c^2/t_D$) and the other with very high mobility ($D=2 \times 10^3 R_c^2/t_D$). The number of deaths is shown in Fig. 6b, where it can be clearly seen how this number is significantly reduced when the focusing strategy is applied.

### City size structure

To generate a population density distribution with a structure which is typical for cities, we add an external potential landscape $U(\mathbf{r})$ to the Langevin equations describing the dynamics of the agents, i.e. $\dot{\mathbf{r}}_i(t)=\sqrt{2D}\varvec{\eta }_i(t) - \nabla _{\mathbf{r}_i} U(\mathbf{r}_i)/\gamma$. Here $\gamma$ is an effective “drag” coefficient determining the strength of the response of the agents to *U*. We now create *U* as a superposition of Gaussians, $U(\mathbf {r})=\sum _j a e^{-\frac{(\mathbf {r}-\mathbf {r}_j)^2}{2\sigma _j^2}}$, each of which leads to a population density maximum around $\mathbf {r}_j$, which represents the center of city *j*. Here *a* is the strength (amplitude) of the reduced potential which we choose as $a=D \gamma /2=kT/2$ and $\sigma _j$ defines the radius of city *j*, which we choose randomly from a distribution $P(\sigma )=\frac{1}{\sigma } \frac{1}{\ln (R_\text{max}/R_\text{min})}$ where $R_\text{min}=20R_c$ and $R_\text{max}=80R_c$ are the minimal and the maximal possible “city radius” in the simulations underlying Fig.4b. We randomly distribute the city centers $\mathbf{r}_j$ within the simulation box.

### Social distancing

To effectively model social distancing in a simple way, we phenomenologically add repulsive excluded volume interactions among the agents which prevent that groups of more than two agents form. That is, we choose $U=\frac{1}{2} \sum _{k,l \ne k} V_{kl} \nu _{kl}$ where the sums run over all agents and where $V_{kl}$ represents the Weeks-Chandler-Anderson interaction potential among agents *k*, *l*, i.e. $V_{kl} = 4\epsilon \left[ (\frac{d}{r_{kl}})^{12} - (\frac{d}{r_{kl}})^6 \right] + \epsilon$ if $r_{kl} \le 2^{1/6}d$ and $V_{kl}=0$ otherwise. Here $r_{kl}$ denotes the distance between agents *k* and *l* and $r_{cut}=2^{1/6} d$ represents a cutoff radius beyond which the interaction potential is zero; $\epsilon$ controls the strength of the potential and is chosen such that $\epsilon /\gamma =D$. In our simulations at each timestep we choose $\nu _{kl}=1$ if at least one of the agent *k* and *l* has a “neighbor” at a distance closer than $d=3R_c$ and otherwise we choose $\nu _{kl}=0$. In addition, we add a weak pair attraction of strength *D*/10 and range $d=3R_c$ to our simulations to support the formation of pairs. That way, agents can form pairs but there is a significantly reduced probability that they form triplets or larger groups.

### Relation of reproduction number to simulation parameters

Here we relate the effective reproduction number $R_e(t)$, which is the average number of infections caused by an infected agent at time *t*, with the microscopic parameters in our simulation. For this purpose, let us first consider the area *A*(*t*) covered by a Brownian agent with radius $R_c$ and diffusion coefficient *D* over a time *t*. This area is known as the Wiener sausage^53^ and reads

3 $$\begin{aligned} A(t) = \pi R_c^2 + \frac{8R_c^2}{\pi } \int _0^{\infty } \frac{1-e^{-\frac{2D y^2 t}{2R_c^2}}}{y^3(J^2_{0}(y)+Y^2_{0}(y))} dy, \end{aligned}$$

where $J_{0}(y)$ and $Y_{0}(y)$ are the 0-th Bessel functions of the first and second kind. Now denoting the agent density of susceptible agents with $\rho _S$, the average number of (possibly infectious) contacts during a time $\tau$ is $A(\tau ) \rho _S$. Thus, if agents are infectious over an overall time of $t_D$ and the fraction of contacts which lead to infections with significant (mild) symptoms is $\beta _r$ ($\beta _o$), we obtain the following expression for the (spatially averaged) effective reproduction number $R_e$:

4 $$\begin{aligned} R_e(t) = A(t_D) \rho _S(t) (\beta _o+\beta _r), \end{aligned}$$

where $R_e(t=0)=R_0$. This expression links the reproduction number with the microscopic simulation parameters and reveals that the reproduction number at time *t* is proportional to the average density of susceptible agents at time *t*.

### Supplementary movie

The movie shows the time-evolution of the modeled infection pattern for $N=55.000$ agents and its response to the proposed spatiotemporal vaccine distribution strategies. Parameters are as in Fig.4b and the population distribution in the movie follows a typical city size structure (Zipf’s law).

## Supplementary information

Supplementary Video.

## Data Availability

The source code of the model has been deposited in a recognized public source code repository (Zenodo, http://doi.org/10.5281/zenodo.4122012).
